# Monocytes and the 38kDa-antigen of mycobacterium tuberculosis modulate natural killer cell activity and their cytolysis directed against ovarian cancer cell lines

**DOI:** 10.1186/1471-2407-12-451

**Published:** 2012-10-04

**Authors:** Nina Gottschalk, Stephan Lang, Rainer Kimmig, Mahavir Singh, Sven Brandau

**Affiliations:** 1 Department of Otorhinolaryngology, University of Duisburg-Essen, Essen, 45147, Germany; 2 Department of Gynecology and Obstetrics, University of Duisburg-Essen, Essen, 45147, Germany; 3 LIONEX Diagnostics and Therapeutics GmbH, Salzdahlumer Straße 196, Braunschweig, 38126, Germany

**Keywords:** NK cell, PstS-1, Ovarian cancer, BCG, Immunotherapy, Cetuximab

## Abstract

**Background:**

Despite strong efforts to improve clinical outcome of ovarian cancer patients by conventional and targeted immuno-based therapies, the prognosis of advanced ovarian cancer is still poor. Natural killer (NK) cells mediate antibody-dependent cellular cytotoxicity (ADCC), release immunostimulatory cytokines and thus function as potent anti-tumour effector cells. However, tumour cells developed mechanisms to escape from an effective immune response. So highly immunogenic substances, like the 38 kDa-preparation of M. tuberculosis, PstS-1, are explored for their potential to enhance cancer-targeted immune responses. In this study we examined the modulation of different NK cell functions by accessory monocytes and PstS-1. We focussed on NK cell activation as well as natural and antibody-dependent cellular cytotoxicity directed against epidermal-growth-factor-receptor (EGFR)-positive ovarian cancer cell lines.

**Methods:**

Activation, cytokine release and cytotoxicity of NK cells stimulated by monocytes and PstS-1 were determined by FACS-analysis, ELISA, Bioplex assay and quantitative polymerase-chain reaction (qPCR). Transwell assays were used to discriminate cell-cell contact-dependent from contact-independent mechanisms. Five ovarian cancer cell lines (A2780, IGROV-1, OVCAR-3, OVCAR-4 and SKOV-3) with different EGFR-expression were used as target cells for natural and antibody-dependent cellular cytotoxicity assays. Cetuximab (anti-EGFR-antibody) was used for ADCC studies.

**Results:**

Our data show that monocytes effectively enhance activation as well natural and antibody-dependent cytolytic activity of NK cells. PstS-1 directly stimulated monocytes and further activated monocyte-NK-co-cultures. However, PstS-1 did not directly influence purified NK cells and did also not affect natural and antibody-dependent cellular cytotoxicity directed against EGFR-positive ovarian cancer cells, even in presence of monocytes. Direct cell-cell contact between NK cells and monocytes was required for NK activation, while released cytokines seemed to play a minor role.

**Conclusions:**

Our data suggest that monocytes enhance natural and antibody-dependent cytotoxic activity of NK cells in a cell-cell contact dependent manner. The TLR-agonist PstS-1 provides additional monocyte activation and induces NK activation markers, while NK cytotoxicity remains unaffected. We conclude that monocytes provide accessory function for ADCC exerted by NK during antibody-based cancer immunotherapy directed against EGFR-positive ovarian cancer cells.

## Background

Ovarian cancer is still the leading cause of death among women with gynaecological malignancies. Despite the primary standard therapy consisting of cytoreductive surgery followed by platinum-taxanes-combined chemotherapy long term survival rates range from 15% to 30% in advanced stages. The addition of further chemotherapeutic agents has not resulted in sufficient clinical benefit so far. Currently immune-based therapies are intensively explored to augment the efficacy of standard oncological treatments. Some immunotherapeutic approaches use non-pathogenic viral or bacterial components as modifiers of the immune response. As an example, BCG (Bacillus Calmette-Guerin), an apathogenic strain of mycobacterium bovis, is a highly effective topic therapy of bladder cancer after initial transurethral tumour resection [1]. This therapy was shown to be superior to local chemotherapy or to the resection of the tumour alone to prevent local recurrence or progression especially in high risk cases [1-3]. Nevertheless, its clinical use is restricted by limited tolerability and the rate of non-responders up to 40% and its absent efficacy against muscle invasive bladder cancer [2,4]. The underlying immunological mechanisms mediating these antitumoural effects are still under investigation, but natural killer (NK) cells supported by accessory monocytes and cytokines seem to play a crucial role [5,6]. More recent data could show that pure BCG is even able to sensitise and activate NK cells directly in absence of antigen-presenting cells (APC) [7].

As an alternative to viable BCG bacteria, the 38 kDa preparation of the cell membrane of mycobacterium tuberculosis, also known as PstS-1, has been developed [8]. PstS-1 is a subunit of the mycobacterial inorganic phosphate uptake system and belongs to the family of ABC (ATP-binding cassette) transporters [9]. In tuberculosis disease PstS-1 is one of the most immunogenic antigens, and the 38 kDa-antigen is therefore included in serodiagnostic assays for active tuberculosis. Further, PstS-1 showed potent immunstimulatory capacity and antitumoural activity in bladder cancer and melanoma [10]. However, in ovarian cancer PstS-1 has not been studied so far. In vitro assays demonstrated stimulating effects of PstS-1 on peripheral blood mononuclear cells (PBMC’s) [10]. In monocytes PstS-1-signals via toll-like-receptors (TLR)-2 and TLR-4 activated ERK1/2 and MAPK-pathways and enhanced the production of IL-6 and TNFα [11,12]. Peptides derived from PstS-1 induced cytolytic activity and the production of IFN-γ in CD8-positive cells [13]. Surprisingly, no data exist on direct or indirect activation of NK cells by PstS-1, although NK cells play a pivotal role in mediating antitumoural effects in immunotherapeutic approaches and might even be directly stimulated by the immunogenic substances [5,7]. In contrast to T-cell immune responses, NK cells are able to mediate anti-tumour activity without prior sensitization to specific tumour antigens. Depending on the expression of CD56 and CD16 human NK cells can be divided into functional subsets: CD16-positive CD56^dim^ NK cells mainly exert cytotoxicity, while CD16-negative CD56^bright^ NK cells are the primary source of immunoregulatory cytokines [14,15]. Cytotoxic NK cells kill target cells via releasing perforin/granzymes or inducing apoptotic pathways like Fas/Fas ligand or TRAIL [16]. For recognising malignant cells the lack of MHC class-I molecules as well as specific receptors on the tumour cell surface (e.g. MIC A/B, ULBPs, B7-H6) are crucial. Corresponding NK cell receptors like NKp46, NKp44, NKp30, and NKG2D are activated dependent on various regulatory receptors such as KIR’s (killer cell immunoglobulin like receptors) and KLR’s (killer cell lectin receptors). In addition to antibody-independent mechanisms cytotoxic NK cells are also able to lyse antibody-coated cells through the FcγRIII (CD16) cell surface receptor exerting antibody-dependent cellular cytotoxicity (ADCC). To achieve their complete effector potential NK cells require activation by cytokines such as IL-15, IL-12, IL-2, TNF and IL-1 mostly released by accessory myeloid cells or T cells [17,18]. Particularly CD16-negative CD56^bright^ NK cells release various cytokines as IFN-γ TNF-α, IL-10, GM-CSF and several chemokines after stimulation and contribute mainly to an immediate effective immune response [15,19]. However, in malignant disease the efficacy of anti-tumour activity of NK cells might be limited since tumour cells have developed several mechanisms to evade NK cell-mediated immunity. Thus, enhancement of NK activity by antibodies and/or immunstimulatory TLR-ligands may improve therapeutic anti-tumour activity of NK cells.

Cetuximab (Erbitux®), the chimeric antibody targeting the EGF-receptor, has been shown to reduce ovarian cancer cell growth and enhance cytotoxic effects of various chemotherapeutic agents in vitro [20]. Since EGFR-overexpression is associated with poor prognosis and is detectable in up to 70% of ovarian cancers targeting the EGF-receptor seemed to be promising [21,22]. However, several clinical studies evaluating cetuximab with or without chemotherapy could not confirm the auspicious preclinical results in vivo [23-25]. Despite the existence of anti-EGFR-resistance in ovarian cancer cetuximab might exert immune modulating effects via antibody-dependent cellular cytotoxicity (ADCC) and might enhance antitumoural activity in a NK cell-based immunotherapeutic approach.

The aim of this study was to examine direct and indirect stimulating effects of monocytes and PstS-1 on secretory and cytotoxic NK cell functions directed against ovarian cancer cells. Established ovarian cancer cell lines with different EGFR-expression and the chimeric anti-EGFR-antibody cetuximab were used as model for ADCC-based immunotherapy.

## Results

### Stimulation of NK cell functions by monocytes

Since monocytes can activate resting NK cells (monocytes-activating effect, MAC) first we evaluated whether accessory monocytes might enhance antitumoural activity of NK cells against ovarian cancer cells. Thus, we determined the expression of CD69 on NK cells and release of IFN-γ in the presence of monocytes and evaluated the cytolytic NK cell activity by the expression of CD107a after additional co-culture with different human ovarian cancer cell lines. In previous studies the ovarian cancer cell lines used (A2780, IGROV-1, OVCAR-3, OVCAR-4 and SKOV-3) had been characterized by flow cytometry for their expression of MHC class I-molecules and the NKG2D-ligand MIC-A. According to these results the five cancer cell lines showed a heterogeneous MHC I-expression with IGROV-1, SKOV-3 and OVCAR-4 displaying a significantly stronger MHC I-expression compared to OVCAR-3 and A2780. MIC-A was consistently expressed in all five cancer cell lines with IGROV-1 exceeding MIC-A-expression of the other cell lines (data not shown).

**Figure 1:**
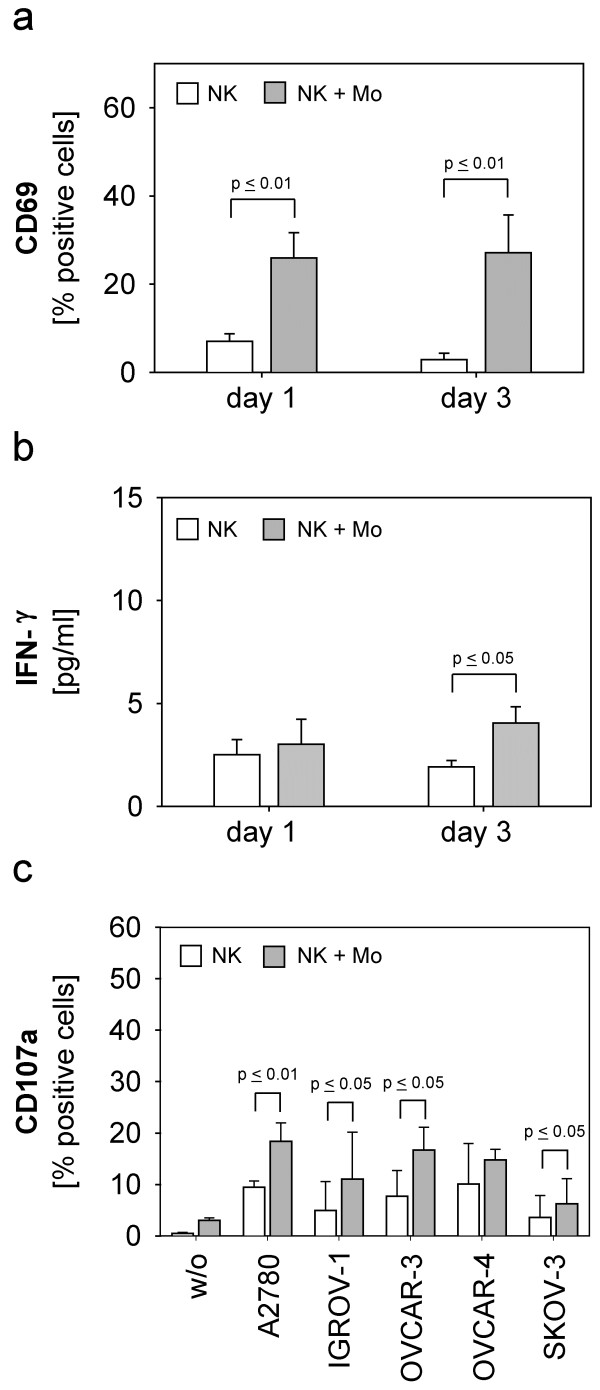
**Stimulation of purified NK cells with monocytes.** Purified monocytes were co-cultured with purified NK cells (5 x 10^5^ cells/well) in 1:1 cell-ratio (NK + Mo) for one and three days. (**a**) Expression of CD69 on NK cells, (**b**) IFN-γ-release and (**c**) cytotoxicity against ovarian cancer cell lines were determined by (**a**) flow-cytometry, (**b**) ELISA and (**c**) CD107a-degranulation-assay, respectively. Data are shown as means + standard error (SE) of three to six independent experiments. Statistical analysis was performed by unpaired t-test, statistical significance (p ≤ 0.05) is indicated.

As illustrated in Figure 1a NK cells showed a statistically significant increase of CD69-positive NK cells on day one (p ≤ 0.01) and three (p ≤ 0.01) in co-culture with monocytes. This MAC-effect also resulted in a slight but significant increase of IFN-γ-release on day three (p ≤ 0.05) **(**Figure 1b**)**. Accordingly, the natural cytotoxicity of NK cells against almost all ovarian cancer cell lines was significantly enhanced by monocyte-stimulation on day one **(**Figure 1c**)**: p ≤ 0,01 for A2780, p ≤ 0,05 for IGROV-1, OVCAR-3 and SKOV-3, respectively. This effect declined on day three due to decreasing cytotoxic NK activity (data not shown).

### Modulation of NK cells by PstS-1 is mediated via monocytes

**Figure 2:**
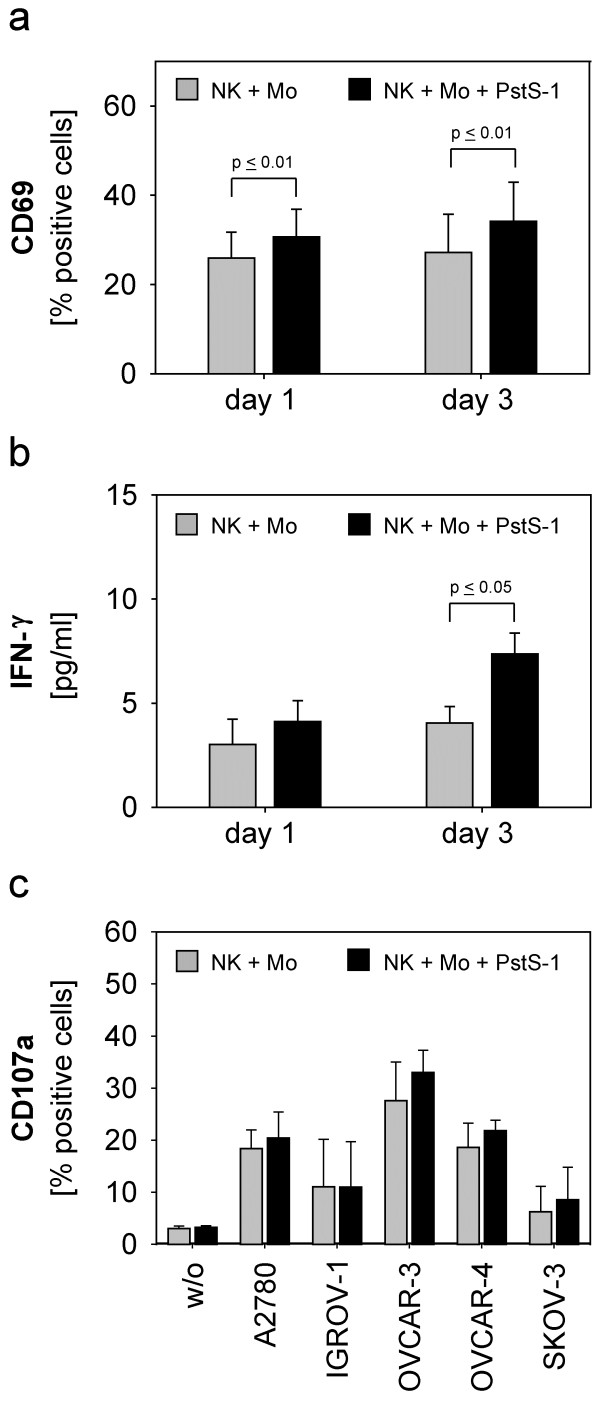
**Stimulation of NK cells in the presence of monocytes and PstS-1.** Purified NK cells (5 x 10^5^ cells/well) were admixed to monocytes (NK + Mo) in 1:1 cell-ratio and cultured with or without PstS-1 (10 μg/ml) for one and three days. NK cells were examined for the expression of CD69 on NK cells by flow cytometry on day one and three (**a**), the IFN-γ-release by ELISA (**b**) and the cytotoxicity against five ovarian cancer cell lines in the CD107a-degranulation-assay on day three (**c**). Means + SE of three to six independent experiments for each cell line are shown. Statistical analysis was performed by unpaired t-test, statistical significance was assumed at p ≤ 0.05.

Based on initial data demonstrating the immunstimulatory potential of PstS-1 on PBMC’s and dendritic cells [10] we addressed the influence of PstS-1 on purified NK cells and monocytes and investigated whether the addition of PstS-1 to the NK-monocyte-co-culture would lead to further enhancement of different NK cell functions. Stimulation of purified monocytes with PstS-1 resulted in a significant dose-dependent release of IL-12 and IL-18 but not IL-15, while surface markers like CD11c, CD80 and CD86 remained unaffected (data not shown). In contrast, isolated NK cells did not respond to direct PstS-1-stimulation, as illustrated by the unchanged expression of CD69 or IFN-γ in response to PstS-1 (data not shown). In NK-monocyte-co-culture PstS-1 could indirectly enhance different NK functions in addition to the MAC-effect described above. Indeed, we found that PstS-1 increased the expression of CD69 on NK cells in the presence of monocytes on day one (p ≤ 0,01) and three (p ≤ 0,01) (Figure 2a) and lead to a slight, significant release of IFN-γ on day three (Figure 2b). However, the cytolytic activity of NK cells directed against various ovarian cancer cell lines could not further be enhanced by PstS-1 (Figure 2c**)**.

**Figure 3:**
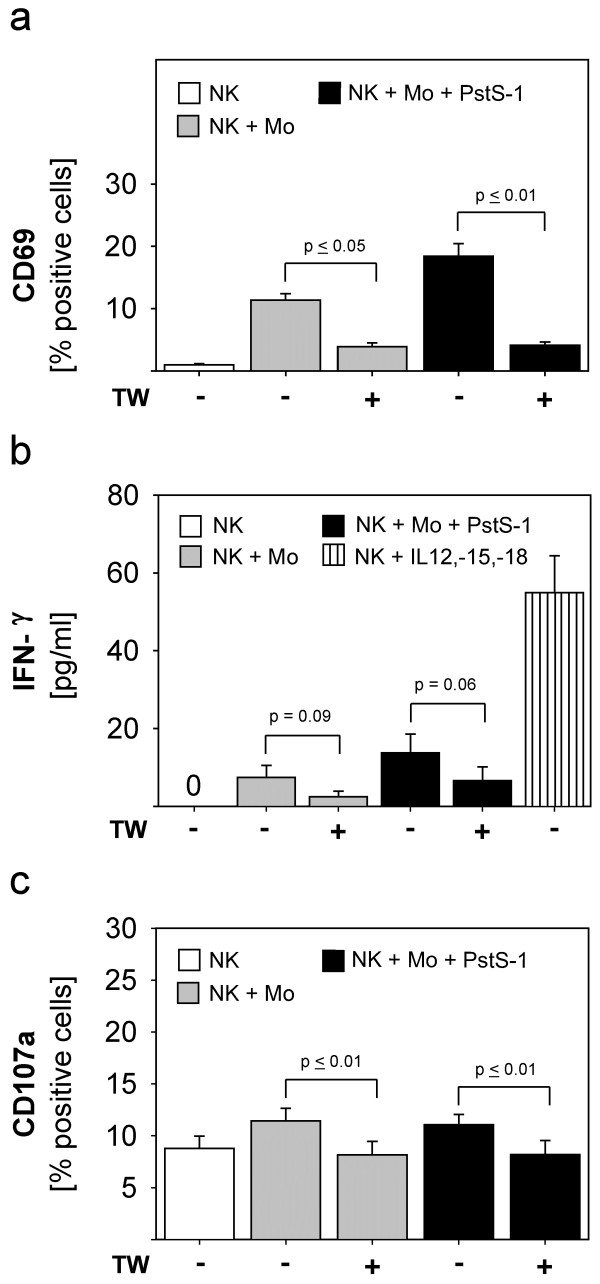
**The role of cell-cell contact during NK cell-stimulation by monocytes and PstS-1.** Purified NK cells (5 x 10^5^ cells/well) were stimulated with monocytes (1:1 cell ratio) with or without PstS-1 (10 μg/ml) for three days. Experiments were performed in presence or absence of a 0,4μm porous membrane (transwell, TW) separating monocytes from NK cells during the stimulation period. CD69-expression on NK cells (**a**), IFN-γ-secretion (**b**) and natural cytotoxicity (**c**) against A2780-cells were determined on day three. Recombinant cytokines (IL-12, IL-15 and IL-18) served as a positive control of IFN-γ-induction (**b**). Means + SE of three to four experiments are shown. Statistical analysis was performed by unpaired t-test, statistical significance was assumed at p ≤ 0.05.

To investigate the mechanisms which might be responsible for the NK-activation by monocytes and PstS-1 we addressed the role of cell-cell contact and cytokines in our model. To this end, we integrated transwells (TW) with a pore size of 0,4 μm to inhibit direct cell-cell contact. As demonstrated in Figure 3a transwells reduced the monocyte-mediated and the PstS-1-enhanced induction of CD69 on NK cells (p ≤ 0,05 and p ≤ 0,01, respectively). Accordingly, the slight but significant induction of IFN-γ by monocytes and additional PstS-1 was also reduced in the absence of cell-cell contact (approached statistical significance: p = 0,09, p = 0,06 respectively). The addition of recombinant IL-12, IL-15 and IL-18 served as a positive control for NK cell activation. Figure 3c shows that the enhanced cytolytic activity of NK cells against A2780-ovarian cancer cells was also reduced to background levels, when cell-cell contact between NK cells and monocytes had been inhibited. These data show that stimulation of NK cells via monocytes and PstS-1 depends on cell-cell contact between NK cells and monocytes.

**Figure 4:**
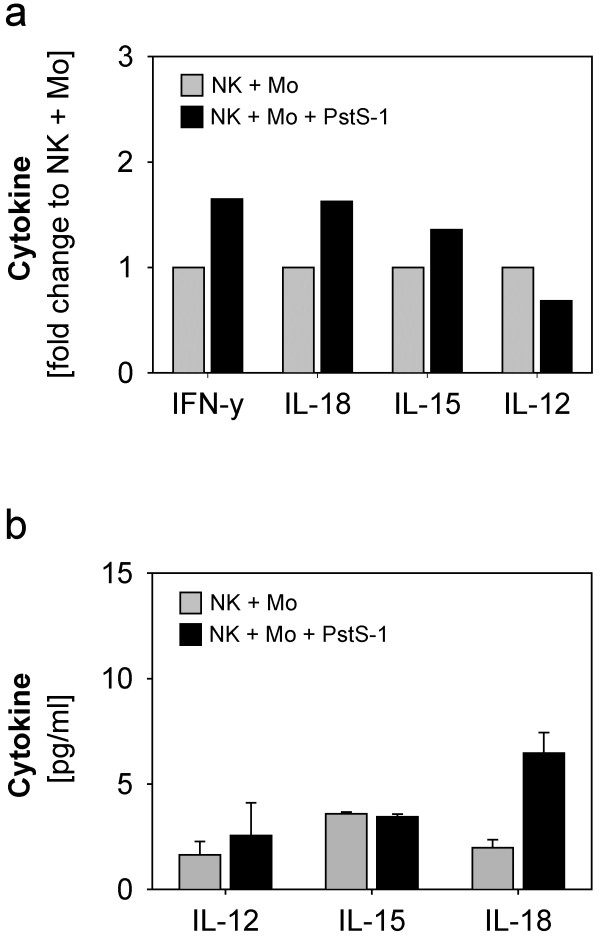
**Cytokine expression in co-cultures of NK cells and monocytes in the presence or absence of PstS-1.** Co-cultures of NK cells and monocytes were stimulated with or without PstS-1 for one and three days. The expression of IL-12, IL-15, IL-18 and IFN-γ was analysed by quantitative RT-PCR. Samples from three independent experiments and donors were pooled. The cytokine expression of the stimulated co-culture (NK + Mo + PstS-1) is shown in relation to the unstimulated (NK + Mo) on day three (**a**). Corresponding to mRNA-expression cytokine protein levels of the samples were determined by BioPlex assay. Cytokine concentrations of the three independent experiments on day three are shown as means + SE in (**b**).

Transwell experiments clearly argued against a role for released soluble cytokines as a mechanism of NK activation in our system. However, as cytokine release is relevant for the modulation of other immune cell subsets in vivo (but not present in our in vitro model), we also determined the expression of selected key cytokines by qPCR (Figure 4a) and Bio-Plex (Figure 4b). Consistent with the IFN-γ-protein levels (Figure 2b) we observed an increased expression of IFN-γ-mRNA due to PstS-1-stimulation on day three (Figure 4a). Expression of mRNA for IL-18 and IL-15 was induced by addition of PstS-1 to NK-monocyte-co-cultures (Figure 4a). However, this transcriptional induction was only translated into a minor augmentation of IL-18 protein release (Figure 4b).

In addition, monocytes co-cultured with NK cells showed a slightly increased CD80-expression in response to PstS-1-stimulation on day one persisting on day three (data not shown). Other surface markers on monocytes (CD86 and CD11c) were not influenced by PstS-1-stimulation (data not shown). In conclusion, NK activation via monocytes and PstS-1 seemed to require direct cell-cell-contact, while released monokines were only of minor relevance.

### Activation of NK cell-subsets

**Figure 5:**
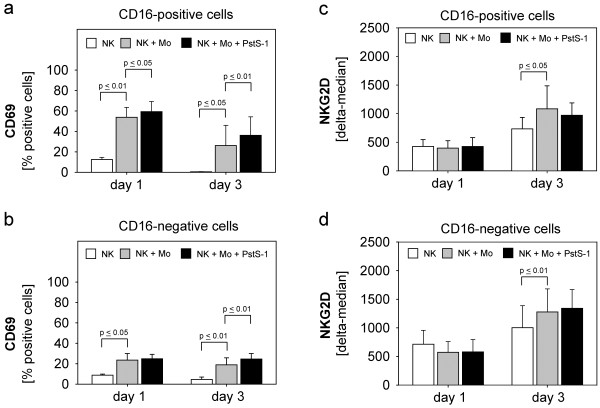
**CD69- and NKG2D-expression of NK cell-subsets.** Isolated NK cells were co-cultured with monocytes with or without PstS-1 as described above for one and three days. NK subsets were discriminated by additional CD16-staining. CD69-expression (**a**+**b**) and NKG2D-expression (**c**+**d**) of CD16-positive- and –negative cells were determined by flow cytometry. Means + SE of five experiments are shown. Statistical analysis was performed by unpaired t-test, statistical significance was assumed at p ≤ 0.05.

CD16-positive NK cells have high cytolytic potential [14], while the CD16-negative NK cell subset produces high amounts of cytokines [15]. We tested whether both subsets are differentially activated in our system. As shown in Figure 5a CD16-positive NK cells are significantly activated by monocytes and additionally by PstS-1 at both time points. Similarly, CD16-negative NK cells were activated by monocytes at both time points (Figure 5b), while the PstS-1-effect was only significant on day three which corresponded to the delayed detection of IFN-γ (Figure 2b and Figure 4a).

Since NKG2D is a main NK cell receptor mediating NK-tumour cell-interaction during cytolysis we investigated whether NKG2D is upregulated by stimulation. As demonstrated in Figure 5c**+**d stimulation with monocytes resulted in a significant upregulation of NKG2D in both NK-subsets on day three, while PstS-1 did not influence the NKG2D-expression.

### Modulation of antibody-dependent cellular cytotoxicity of stimulated NK cells directed against human ovarian cancer cell lines

**Figure 6:**
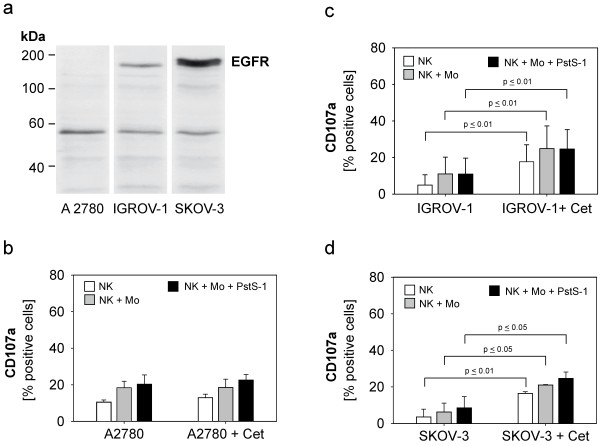
**Antibody-dependent cellular cytotoxicity (ADCC) of NK cells directed against ovarian cancer cell lines.** Total lysates (20 μg) of each cell line were separated in a 7.5 %-SDS-polyacrylamid gel, immunoblotted with primary anti-EGFR antibody followed by peroxidase-labelled secondary antibody and visualised by chemiluminescence. (**a**) The expression of total EGFR (170 kDa) in three ovarian cancer cell lines are shown. The band at 53–55 kDa most likely reflects cross-reactivity of the antibody with EGFR-related protein (ERRP). (**b**-**d**) The ovarian cancer cell lines A2780, IGROV-1 and SKOV-3 were co-cultured with cetuximab (1 μg/ml) and NK cells, which were stimulated by monocytes with and without PstS-1 (NK + Mo + PstS-1) for three days. CD107a-degranulation-assay. Data are shown as means + SE of three to six independent experiments on day three. Statistical analysis was performed by unpaired t-test, statistical significance was assumed at p ≤ 0.05.

Based on the apparent enhancement of natural NK cytotoxicity by monocytes, we evaluated a potential modulation of antibody-dependent cellular cytotoxicity directed against different ovarian cancer cell lines. For targeting of the EGF-receptor we used the chimeric antibody cetuximab and the cell lines A2780, IGROV-1 and SKOV-3. The EGFR-expression of these ovarian cancer cell lines was analysed by western blotting **(**Figure 6a**)**. The cell line A2780 did not express EGFR and served as a negative control in our experiments. IGROV-1 and the SKOV-3-cell line showed intermediate and strong EGFR-expression, respectively. All three ovarian cancer cell lines were coincubated with unstimulated and monocyte-stimulated NK cells with or without PstS-1. Cetuximab was added in a concentration of 1 μg/ml, which has been titrated in preliminary experiments (data not shown). As expected, in the EGFR-negative cell line A2780 adding cetuximab did not result in any additional ADCC-activity. In contrast, the EGFR-positive cell lines IGROV-1 and SKOV-3 showed a strong ADCC-sensitivity mediated by cetuximab which reached statistical significance (IGROV-1: p ≤ 0,01, SKOV-3: p ≤ 0,01 and p ≤ 0,05 respectively). Cetuximab-mediated ADCC was significantly enhanced by accessory monocytes but not PstS-1.

In summary, we showed that monocytes strongly augmented various NK functions including the activation of ADCC against ovarian cancer targets. Some, but not all NK functions could be further enhanced by the addition of the immunostimulatory mycobacterial agent PstS-1. Activation of NK cells was strongly dependent on cell-cell contact to monocytes.

## Discussion

In immunotherapeutic approaches viral or bacterial components are used to augment immune cell responses to tumour cells. For example, viable BCG showed clear antitumour activity in clinical trials [1,7]. Mechanistic studies demonstrated that activation of NK cells may contribute to this efficacy [5,6]. The alternative 38kDa-preparation of M. tuberculosis, PstS-1, has been shown to effectively stimulate PBMC’s and mediate anti-tumour activity [10]. Because of these promising preliminary results, PstS-1 might be a suitable enhancer for future immunotherapeutic approaches. In this study we tested the capacity of PstS-1 to stimulate NK cells and to induce NK activity directed against ovarian cancer cells.

Our initial results showed that monocytes efficiently stimulated antitumoural NK cell functions efficiently (MAC-effect). The CD16-positive NK-subset was significantly activated by monocytes (upregulation of CD69) which is likely to explain the enhanced natural cytotoxicity against various ovarian cancer cells. Transwell-experiments revealed that direct cell-cell contact between NK cells and monocytes was indispensible for NK-stimulation, while soluble factors seemed to be of minor importance. Our results are consistent with other data, which showed that monocytes could potentially activate NK cells and were crucial in enhancing NK cell-mediated cytotoxicity against tumour cells [6]. Clearly, cytokines like IL-12, IL-15 or IL-18 derived from monocytes have the potential to stimulate NK cells [26]. Similar to our data, it has also been shown that direct cell-cell contact between NK cells and monocytes is crucial for NK activation [27]. These data are further supported by studies regarding interactions between dendritic cells and NK cells which also emphasized the essential role of direct cell-cell contact [28]. Our studies could further show that monocytes lead to upregulation of the NKG2D-receptor on CD16-positive as well as CD16-negative NK cells. Since NKG2D is a main receptor for tumour-NK cell-interaction during the cytolysis, monocyte-mediated upregulation of NKG2D might play a role in monocyte-induced cytotoxicity of NK cells. From these data we conclude that monocytes potently enhance NK-cytotoxicity directed against ovarian cancer cells.

Our own studies revealed that PstS-1 stimulated monocytes. This is consistent with other data showing that PstS-1 stimulates monocytes via TLR-2 and TLR-4 involving ERK1/2- and MAPK-pathways [11]. Additionally, PstS-1-stimulation was followed by substantial cytokine-release [11], which could be confirmed in our study. In contrast, PstS-1 remained without any direct effect on purified NK cells illustrated by lack of CD69-expression and absent IFN-γ-induction. Although direct activation by pathogen-derived molecules is not a classical feature of NK cells, some recent studies suggest that NK cells might also directly recognize bacterial components. For example, BCG could stimulate NK cells in absence of APC [7]. Other data indicate that different TLR’s like TLR-2 and TLR-5 on NK cells might be involved [29]. However, in our model, PstS-1 activated NK cells only in the presence of accessory monocytic cells. In NK-monocyte-co-cultures PstS-1 enhanced monocyte-mediated NK activation illustrated by a further significant increase of CD69-expression. In these experiments both the CD16-positive and the CD16-negative NK-subset were activated. However, the only minimal induction of IFN-γ in our system indicated that the activation of the CD16-negative subset did not result in substantial induction of cytokine release by this subset. This is in contrast to other data demonstrating that BCG-stimulated monocytes can induce substantial IFN-γ-release in NK cells [6]. Direct cell-cell contact with monocytes was crucial for NK activation in absence or presence of PstS-1, while monokines (IL-12, IL-15 and IL-18) seemed to play a minor role. Although the CD16-positive NK-subset in co-culture with monocytes was activated by PstS-1, natural cytotoxicity against various ovarian cancer cells remained largely unaffected by PstS-1. This is in contrast to previous data, which showed that dendritic cells are activated by PstS-1 [10] and by studies indicating enhanced NK cytotoxicity against bladder cancer cells in BCG-stimulated mononuclear cells [7,10]. However, in accordance to our data, Kloss et al. demonstrated that monocytes stimulated by the TLR4-agonist LPS increased IFN-γ-production in NK cells but did not to enhance cytotoxicity to target cells [30]. In conclusion, PstS-1 stimulates selected NK functions via monocytes activation, while NK cytotoxicity seems to be unaffected by PstS-1.

Our studies showed that ovarian cancer cells are mainly resistant to NK-mediated cytolysis. Thus, we studied whether NK-susceptibility can be increased by addition of the anti-EGF-receptor-antibody cetuximab mediating antibody-dependent cellular cytotoxicity and further enhanced by additional co-cultured monocytes and PstS-1. The EGFR-positive cell lines IGROV-1 and SKOV-3 displayed a significant enhanced susceptibility to NK cytolysis in presence of cetuximab while, as expected, the EGFR-negative cell line A2780 showed no ADCC-effect. Monocytes could further enhance the ADCC-mechanism, while PstS-1 remained without any effect. Our data support other studies which could demonstrate that ovarian cancer cells are highly resistant to natural cytolysis but might respond to ADCC-mechanism-based immunotherapy [31]. Especially in chemoresistant ovarian cancer this therapeutic approach could be valuble [31]. Thereby, EGFR might be a suitable target since EGFR is overexpressed in up to 70% of ovarian cancer [21] and anti-EGFR-antibodies like cetuximab and panitumumab are tested in clinical setting. Thus, our study suggests that antibody- and NK-based immunotherapies are functionally supported by monocytes in ovarian cancer.

## Conclusions

In summary, our data demonstrated an accessory function of monocytes during both antibody-dependent and –independent anti-tumour cytotoxicity of NK cells. The immunstimulatory bacterial agent PstS-1 effectively stimulated monocytes and further induced selected NK functions.

## Methods

### Cell culture and cell lines

The human ovarian cancer cell lines IGROV-1, SKOV-3 and OVCAR-3 were obtained from Dr. M. Mallmann, MD (*Life and Medical Sciences Institute Bonn*, University of Bonn, Germany), the ovarian cancer cell lines A2780 and OVCAR-4 were provided by *Westdeutsches Tumorzentrum*, University of Duisburg-Essen, Germany. IGROV-1, SKOV-3 and OVCAR-3 were cultured in standard medium RPMI-1640 (Invitrogen, Karlsruhe, Germany) supplemented with 10% fetal calf serum (FCS, Biochrom, Berlin, Germany), 100 units/mL penicillin and 100 μg/mL streptomycin (Invitrogen, Karlsruhe, Germany). A2780 and OVCAR-4 were cultured in modified medium consisting of RPMI-1640 and DMEM (high-glucose Dulbecco’s Modified Eagle Medium, Invitrogen) (3:1 vol/vol) supplemented with 10% FCS (Biochrom), 1% penicillin/streptomycin (Invitrogen) and 1% sodium pyruvate (Invitrogen). Tumour cells were incubated in plastic culture flasks (Greiner, Solingen, Germany) at 37°C and 5% CO_2_ and continuously passaged by treatment with Accutase (PAA, Pasching, Austria) for 5 minutes at 37°C.

### Isolation of NK cells and monocytes from PBMC’s of healthy donors

Blood samples (50–150 ml) of healthy donors were obtained in citrate monovettes (Sarstedt AG & Co., Nümbrecht, Germany) and diluted (1:1, vol/vol) with phosphate-buffered saline (PBS) and separated by density centrifugation (Biocoll Separating Solution, Biochrom AG, Berlin, Germany) at 25°C, 300g for 30 minutes. The mononuclear cell (MNC) fraction was collected, washed repeatedly with PBS and counted (CasyCounter; Innovatis-Roche, Bielefeld, Germany). For further isolation of NK cells and monocytes the magnetic cell separator NK isolation kit II and CD14-beads (Miltenyi Biotec, Mönchengladbach, Germany) were used according to manufacturer’s protocol. The separated immune cells were used for experiments immediately after isolation. Freezing of NK cells at −20°C resulted in a significant loss of activity. Therefore, all experiments in this study were performed with freshly isolated and purified NK cells. Purity of cell subsets was routinely tested and ranged from 90% to 97%.

### Stimulation of purified NK cells and monocytes

Purified NK cells and monocytes, single or in co-culture (0.5 x 10^6^ cells/well, cell-cell-ratio 1:1) were stimulated with 10 μg/ml of PstS-1 (obtained from M. Singh, PhD, Lionex GmbH, Braunschweig, Germany) in a 24 well plate (Greiner Bio-One, Frickenhausen, Germany). In some experiments 10mm Tissue Culture Inserts with 0,4 μm Anapore® Membrane (NUNC, Roskilde, Denmark) were inserted during stimulation to inhibit cell-cell contact between monocytes and NK cells. CD69- and NKG2D-expression on NK cells and the expression of CD80, CD86 and CD11c on monocytes were determined on day one and three of stimulation. NK subsets were differentiated by the addition of anti-CD16. Supernatants were collected for cytokine-analysis by ELISA and BioPlex assay.

### Human IFN-γ- and IL-18-ELISA

The supernatants of stimulated NK cells and unstimulated controls were recovered over stimulation time (one and three days) and examined for the presence of IFN-γ, IL-12, -15 and −18. Detection of IFN-γ was performed with an anti-human IFN-γ-ELISA-kit (R&D Systems, Wiesbaden, Germany), IL-18 was detected by an IL-18-ELISA kit (Benders MedSystems (eBioscience, San Diego, USA). Both kits were used according to manufacturer’s protocol.

### BioPlex assay for IL-12 and IL-15

For the detection of IL-12 and IL-15 a multiplex immunoassay (BioPlex assay) was used. Magnetic microspheres dyed with two fluorochromes and conjugated with specific monoclonal antibodies against the target protein were used according to the manufacturer’s instructions (Millipore Corporation, Billerica, USA). Each experiment was performed in duplicate using a 96-well plate. The analytes concentration was determined from the standard curve by analysis of mean fluorescent intensity values using a Bio-Plex array reader (Bio-Rad, Laboratories, Hercules, CA) with software (Bio-Plex Manager™ 6.0 Software).

### Flow cytometric analysis (FACS)

For FACS-analysis the following antibodies were used: anti-CD107a-FITC (clone H4A3), anti-CD86-RPE (clone 2331 FUN-1), anti-CD11c-APC (clone B-ly6), anti-CD16-PE-Cy7 (clone 3G8) and all from BD Bioscience, Heidelberg, Germany. Anti-CD80-PE (clone MAB104) and anti-CD69-FITC (clone FN50) from Invitrogen, anti-CD14-PerCP/Cy5.5 (clone HCD14) from BioLegend GmbH, Fell, Germany, anti-NKG2D-RPE from R&D Systems, anti-MICA (clone AMO1) from Immatics, Tübingen, Germany, anti-HLA-classI-RPE (clone W6/32) from Dako. Corresponding isotypes were used as controls. Cells were analysed on a FACS Canto II using Diva software 6.0 (Becton Dickinson, Heidelberg, Germany).

### CD107a degranulation assay

Since the lysosomal-associated membrane protein-1 (LAMP-1 or CD107a) in NK cells is expressed during degranulation and correlates with NK cell-mediated tumour cell lysis [32] the expression of CD107a on NK cells was used to evaluate natural and antibody-mediated NK cell cytotoxicity. NK cells stimulated with monocytes with or without PstS-1 for one and three days were seeded on a flat-bottom 96-microtiter well plate (Greiner Bio-One). Tumour cells of the ovarian cancer cell lines A2780, IGROV-1, OVCAR-3, OVCAR-4 and SKOV-3 were coincubated in 1:1 cell ratio. The monoclonal anti-EGFR-antibody Cetuximab (Erbitux®, ImClone Systems, Bristol-Myers Squibb, New York, USA und Merck KGaA, Darmstadt, Germany) concentrated 1 μg/ml was directly added in experiments evaluating the ADCC of NK cells. Samples without antibody and unstimulated NK cells served as controls. NK cells were labelled with anti-CD107a-FITC or isotype mIgG1-FITC 1:20, the golgi-stop monensin (BD Golgi-stop, BD Bioscience) was added 1:600 after one hour incubation at 37°C in 5% CO_2_. After 5 hour incubation at 37°C in 5% CO_2_ cells were resuspended in 200 μl azide-PBS and immediately analysed in the flow cytometer.

### RNA isolation and quantitative polymerase chain reaction

For quantitative polymerase chain reaction (qPCR) of IFN-γ, IL-12, IL-15 and IL-18 mRNA from co-cultured NK cells and monocytes stimulated with or without PstS-1 for one and three days were isolated using RNeasy Mini kit (Qiagen, Hilden, Germany) according to the manufacturer’s protocol. RNA concentrations and purity were determined at 260 nm/280 nm photometrically. For cDNA-synthesis the RNA of three donors and independent experiments was pooled and reverse-transcribed using Super Script II RNase H- Reverse Transcriptase kit (Invitrogen) and hexamer-random primer (Invitrogen) according to manufacturer’s protocol. The cDNA was used as template for the quantitative real-time PCR (Light cycler 2.0, Roche Diagnostics GmbH, Mannheim, Germany) using specific primer for IFN-γ, IL-12, IL-15 and IL-18 (Invitrogen). The following sets of primers were used: IFN-γ sense 5^′^-GAGTGTGGAGACCATCAAGGAAG-3^′^ and antisense 5^′^-TGCTTTGCGTTGGACATTCAAGTC-3^′^, IL-12A(p35) sense 5^′^-TGCCTTCACCACTCCCAAAACC-3^′^ and antisense 5^′^-CAATCTCTTCAGAAGTGCAAGGG-3^′^, IL-15 sense 5^′^-AACAGAAGCCAACTGGGTGAATG-3^′^ and antisense 5^′^-CTCCAAGAGAAAGCACTTCATTGC-3^′^, IL-18 sense 5^′^-GATAGCCAGCCTAGAGGTATGG-3^′^ and antisense 5^′^-CCTTGATGTTATCAGGAGGATTCA-3^′^. The conditions for amplification were as follows: initial denaturation at 95°C for 10 minutes, followed by 50 cycles of 10 s at 95°C, 20 s at the primer-specific temperature of 60°C, 20s at 72°C for extension. The amplified PCR products were separated by 2% agarose gel electrophoresis and visualised after ethidium bromide staining.

### Western blot

Western blot analysis for the determination of the EGFR-expression of various ovarian cancer cell lines was performed. Protein of total lysates (20 μl of each cell lines) were separated by SDS-7.5%-polyacrylamide gel and immunoblotted according to semi-dry-blot-method onto polyvinylidene difluoride membrane (PVDM, Roche Diagnostics). The membrane was incubated with the monoclonal primary antibody anti-EGFR (BioLegend) followed by AP-conjugated secondary goat-anti-rabbit IgG. Bands were visualised after application of the substrate CDP-star (Roche Diagnostics) and chemiluminescent transformation. The chemiluminescent signal was recorded with ChemiDoc-It Imaging system (UVP, Upland, California, USA) after exposure time of 20 minutes.

### Statistical analysis

Data are presented as mean and standard error of several independent experiments. For statistical evaluation unpaired t-test was performed assuming statistical significance level of p ≤ 0.05. The statistical calculations and illustrations were performed using SigmaPlot for Windows Version 11 (Systat Software GmbH, Erkrath, Germany).

## Competing interests

Lionex GmbH manufactured and provided purified PstS-1 for this study. MS is CEO of Lionex GmbH. No conflict of interest is caused by this fact. The other authors also declare to have no conflict of interests.

## Authors’ contributions

NG carried out and designed all experimental studies and wrote the manuscript. SL was responsible for clinical management. RK edited the manuscript. MS purified and provided PstS-1 after quality testing. SB conceived of the study, designed experiments and wrote the manuscript. All authors read and approved the final manuscript.

## Pre-publication history

The pre-publication history for this paper can be accessed here:

http://www.biomedcentral.com/1471-2407/12/451/prepub
